# Fluorescence lymphography in kidney transplantation (FLIKT-study)—real time intraoperative lymphography to avoid lymphocele formation

**DOI:** 10.1007/s00464-026-12656-1

**Published:** 2026-03-04

**Authors:** M. Hummels, T. Wagner, D. Buchner, A. R. Stier, R. Datta, L. Ischebeck, H. F. Fuchs, M. Kann, F. C. Koehler, C. Kurschat, C. Gietzen, J. Becker, C. J. Bruns, D. L. Stippel, M. N. Thomas

**Affiliations:** 1 Department of General-, Visceral-, Thoracic- Und Transplantation Surgery, University of Cologne, Kerpenerstr. 62, 50937 Cologne, Germany; 2 Department II of Internal Medicine and Center for Molecular Medicine Cologne, Faculty of Medicine and University Hospital Cologne, University of Cologne, Cologne, Germany; 3 Cologne Excellence Cluster on Cellular Stress Responses in Aging-Associated Diseases (CECAD), Faculty of Medicine and University Hospital Cologne, University of Cologne, Cologne, Germany; 4 Department of Internal Medicine (Nephrology) & Einthoven Laboratory of Vascular and Regenerative Medicine, Leiden University Medical Center, Leiden, the Netherlands; 5 Department of Radiology, University of Cologne, Cologne, Germany; 6 Institute of Pathology, University Hospital of Cologne, Cologne, Germany

**Keywords:** Lymphocele, Kidney transplantation, ICG, Lymphography

## Abstract

**Background:**

Lymphocele formation is a frequent complication after kidney transplantation, occurring in up to 26% of recipients. It results mainly from lymphatic injury during dissection of the iliac vessels and can impair graft function by compressing the renal graft, vascular structures, or ureter. Improved intraoperative visualization of lymphatic vessels may reduce this risk. Indocyanine green (ICG) fluorescence technology enables real-time lymphatic mapping. This study evaluated the feasibility and clinical utility of ICG fluorescence-guided lymphography during kidney transplantation.

**Methods:**

In a prospective single-center study, ICG lymphography was performed in 21 consecutive living-donor kidney transplantations. Under ultrasound guidance, ICG was injected into the subcutaneous tissue of the ipsilateral femoral triangle one hour before surgery. Lymphatic structures were visualized intraoperatively using a handheld fluorescence imaging system (Spy-Phi, Stryker) at four procedural time points. Identified lymphatic vessels were preserved, and any visualized lymphatic leakage was clipped. Postoperative surveillance included weekly ultrasonography in the early postoperative phase and MRI at 6 months.

**Results:**

ICG injection enabled clear visualization of retroperitoneal lymphatic vessels in all 21 cases. Lymphatic structures were successfully preserved during dissection. In two cases, intraoperative lymphatic leakage was detected and controlled. Postoperative ultrasound showed no perirenal fluid collections in any patient. MRI performed in 15/21 patients at 6 months confirmed absence of lymphocele formation.

**Conclusion:**

ICG-guided lymphography is a safe, feasible technique for intraoperative visualization of lymphatic vessels during kidney transplantation and may help prevent lymphocele formation.

The occurrence of lymphoceles in kidney transplantation (KTX) is one of the most common complications and is described in the literature with an incidence of 6%-40%[1–4]. It is defined as the accumulation of lymph fluid within a non-epithelialized cavity surrounded by pseudomembranes around a graft [3]. However, the reasons for lymphocele formation remain under discussion. Lymph fluid may accumulate from lymph secretions due to extensive dissection of the lymphatic vessels accompanying the iliac vessels during vascular anastomosis or iliac dissection or from the transplanted kidney itself [5]. Radioisotope studies, in turn, have suggested that most lymphoceles originate from the un-ligated iliac lymphatic vessels of the recipient [6]. While most lymphoceles occurring after KTX remain asymptomatic, the incidence of lymphocele is reported 6 weeks after KTX [6].

Most lymphoceles are small, asymptomatic, and incidentally diagnosed during follow-up ultrasonography. However, depending on the location or size, lymphoceles can become symptomatic or even negatively impair kidney function due to compression of adjacent organs or the graft itself [7]. The most common secondary complications of lymphoceles are ureteral compression and compression of vascular structures, leading to venous thrombosis or visceral compression and abdominal discomfort or wound dehiscence.

The etiology of lymphoceles is multifactorial. Risk factors include extensive lymphatic dissection, inadequate ligation of lymphatic channels during back-bench preparation of the renal hilum, use of mTOR inhibitors such as sirolimus and everolimus, ABO-incompatible kidney transplantation, recipient age, sex and body mass index, dialysis time, episodes of acute rejection, and high doses of corticosteroids and anticoagulants [8]. Diagnosis is generally made by ultrasonography, mostly combined with fluid aspiration and biochemical analysis of the aspirate, ruling out a urine leak or urinoma.

Most small asymptomatic lymphoceles resolve spontaneously, and no further therapeutic management is necessary. For those that require treatment, the first diagnostic and therapeutic step is the ultrasound-guided aspiration of fluid collection. However, the recurrence rate is as high as 95%. A combination of fluid aspiration with the administration of sclerosing agents (povidone-iodine, human fibrinogen, and streptomycin) showed recurrence rates of 50% [9–11]. Therefore, the treatment of choice for symptomatic lymphoceles remains surgical marsupialization, which allows the lymphatic fluid to drain into the peritoneal cavity. Here, a recurrence rate of 8% was achieved [12].

Given their potential to adversely affect graft function and patient morbidity, lymphocele prevention is the most important goal in both surgical techniques and post-transplant care. In this context, continued refinement of surgical techniques is essential to reduce the incidence and improve transplant outcomes.

Indocyanine green (ICG) fluorescence imaging systems are promising complementary techniques in various surgical fields, including visualization of biliary/vascular anatomy in laparoscopic surgery or identification of tumor tissue in oncological liver surgery and sentinel lymph nodes [13–15]. ICG can also be used for lymphography and intraoperative localization of lymphatic fistulas. In addition, Ietto et al. [16] demonstrated in a case report that real-time ICG lymphography during kidney transplantation is feasible and results in clear visualization of lymphatic vessels during iliac dissection during kidney transplantation.

Therefore, we conducted this prospective pilot study in a larger, controlled cohort of living donation kidney transplant recipients with the goal of visualizing and preserving adjacent lymphatic tissue during the transplant procedure and preventing the formation of lymphoceles in the posttransplant period.

## Materials and methods

The institutional review board approval for this prospective cohort study was authorized by the Ethics Committee of the Medical Faculty of the University of Cologne (No.: 21-1292_1), and the study was conducted at the surgical department of the University of Cologne, Germany, between 11/22 and 08/23. Living kidney transplantation was performed as described previously in 21 consecutive kidney transplant recipients [17]. The only exclusion criterion was allergy to ICG dye, which was never encountered during the study. Patient information and written informed consent were obtained from all patients enrolled in this study for ICG lymphography and MRI during the follow-up.

**Fig. 1:**
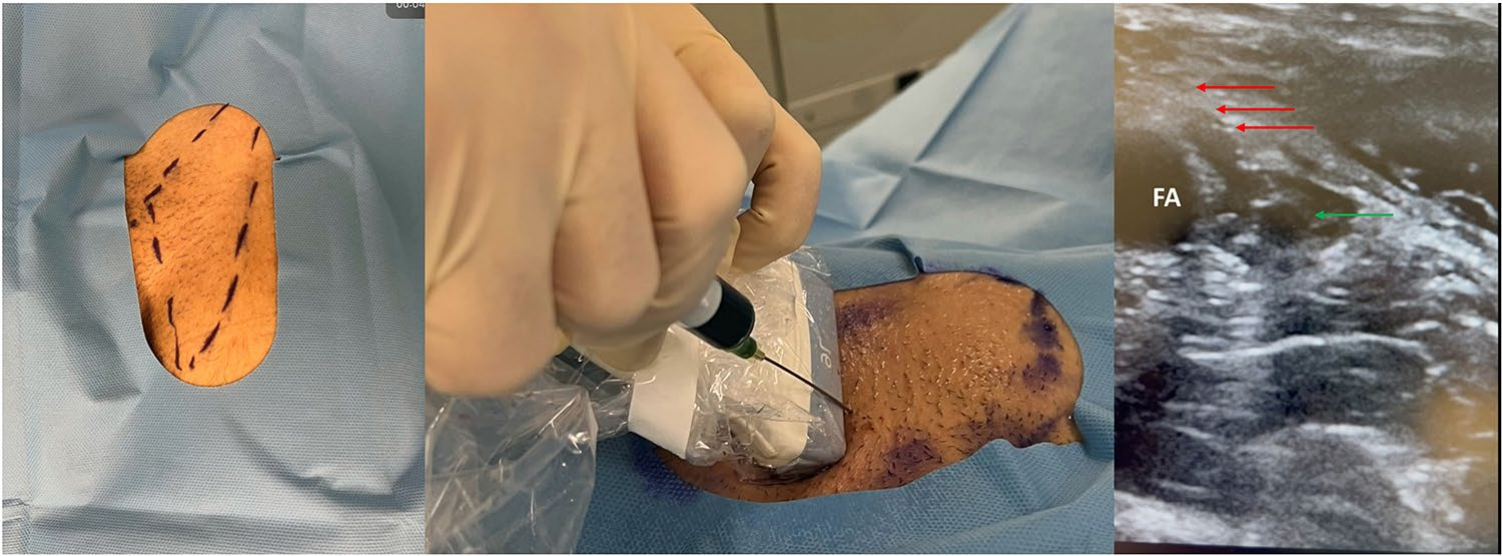
ICG injection in the right groin (Femoral artery = F, track of the needle = red arrows, injection side and collection of ICG = green arrow)

ICG (Verde Indocianina PULSION 25 mg/50 mg; PULSION Medical Systems, Feldkirchen, Germany) was prepared by dissolving 25 mg sterile ICG in 10 ml of saline. One hour before skin incision and during induction of general anesthesia, 2 ml of the prepared ICG solution was injected subcutaneously into the Scarpa´s triangle using a 20-gauge needle, under sonographic control, directly next to the femoral artery. Immediately after the injection, manual pressure was applied to the injection side to facilitate ICG resorption (Fig. 1).

All ICG measurements were conducted in living-donor kidney transplantation. The demographic data of the kidney donors are shown in Table 1. Donor nephrectomy was performed using the HARP technique, as first described in 2002 by Wadström et al.[18], by an experienced surgeon (> 500 consecutive HARP donor nephrectomies). For preparation and dissection, Caiman 5 Articulating (B. Braun, Melsungen, Germany) was used in all cases. In each case, a pre-implantation kidney biopsy for histopathological evaluation of the donor kidney was performed as part of our center’s routine. No biopsies were missing. The mean glomerular count was 31 (22–49; standard deviation: ± 10). Eleven patients had moderate atherosclerosis (Banff Lesion Score cv2), and one patient had severe arteriosclerosis in the pre-transplant biopsy (Banff Lesion Score cv3). Eight patients had sclerotic glomerula (3–5 in the biopsy sample) (Table 1).

**Table 1:** Living kidney donation donor characteristics, shown in mean and range (brackets)

Sex (W/M)	11/10
Age (a)	60 (40–80)
Height (cm)	170 (155–190)
Weight (kg)	80 (52–108)
BMI (kg/m^2^)	25,8 (20–35)
Donor kidney volumetry (ccm)	82,7 (51–128)
Warm ischemic time (sec)	139 (92–244)
Total duration of surgery (min)	113 (77–181)
Side of donation (l\r)	12/9
Multiple arteries (y/n)	7/14

Kidney transplantation was performed according to the standard protocol of the transplant center. Iliac vein transposition was performed in every case, resulting in the thorough mobilization of the external iliac veins and arteries. Before anastomosis, the internal iliac veins were dissected to elevate the axis of the iliac vein and facilitate venous anastomosis. The demographic data of the kidney recipients are summarized in Table 2.

**Table 2:** Living donation kidney recipients characteristics, shown in mean and range (brackets)

Sex (W/M)	10/11
Age (a)	42 (23–74)
Height (cm)	174 (158–192)
Weight (kg)	80,5 (53–120)
BMI (kg/m^2^)	26,8 (21–37)
Cause of kidney failure (*n*)	
Glomerulonephritis	15
Autosomal dominant polycystic kidney disease	2
Diabtec nephropathy	2
unknown	2
Total duration of surgery (min)	149 (115–235)
Warm ischemic time (min)	32,5 (25–67)
Cold ischemic time (min)	184 (158–266)
Complications Clavien-Dindo > 3a (n)	0
Creatinine serum level discharge (mg/dl)	1,58 (0,73–3,04)

**Fig. 2:**
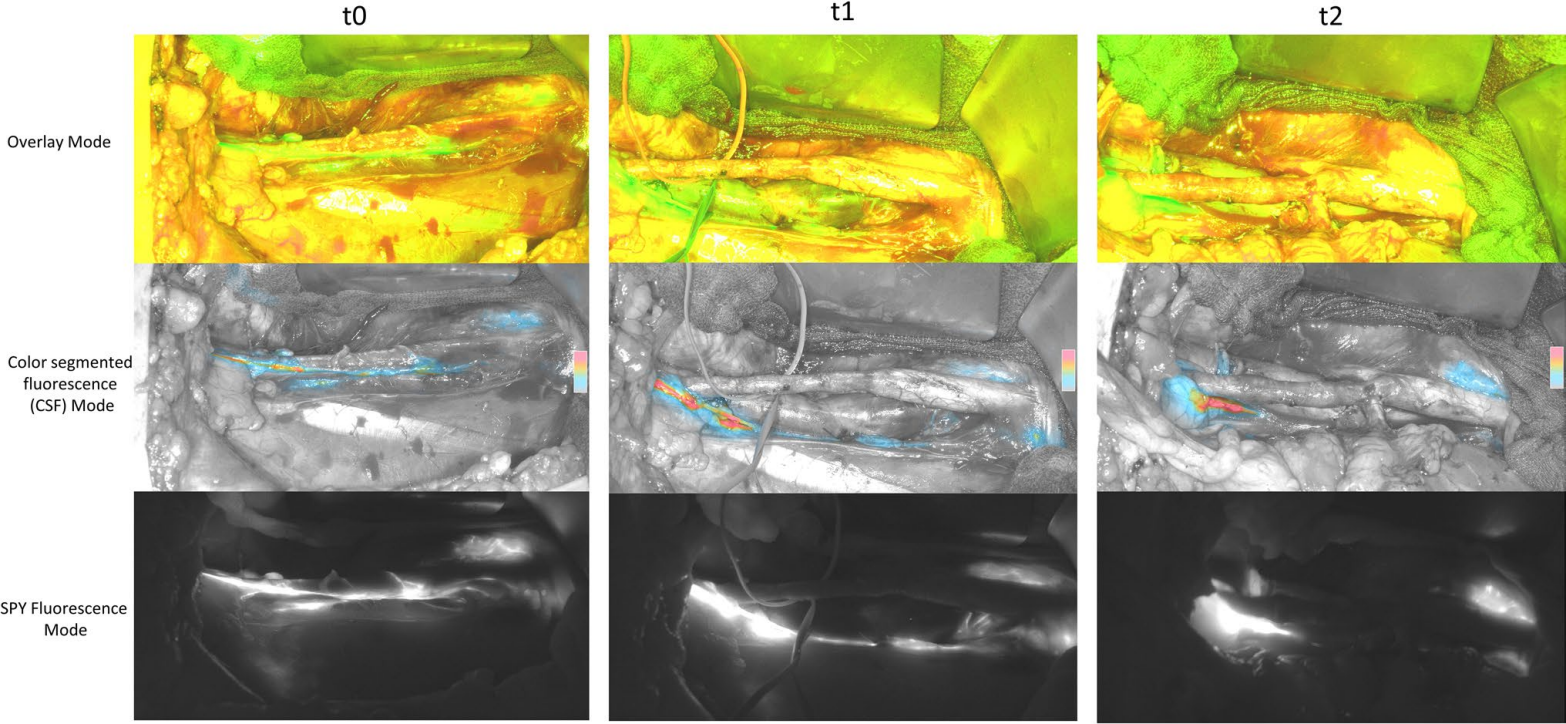
Intraoperative fluorescence lymphography at t0, t1, and t2 during kidney transplantation showing the 3 different fluorescence modalities (Overlay mode, color segmented fluorescence mode, and SPY Fluorescence mode)

Four intraoperative fluorescence imaging time points were defined during the transplant procedure using the Spy Portable Handheld Imager (SPY-Phi, Stryker, Portage USA). The first measurement was carried out at the beginning of iliac preparation (t0) in order to visualize the lymphatic vessels, the second measurement before starting the kidney transplant procedure and vascular anastomosis in order to visualize the integrity of the lymphatic vessels (t1), the third measurement 5 min after reperfusion of the transplanted kidney (t2), and the fourth measurement before wound closure (t3) (Fig. 2). At every time point, three images were recorded using the different modalities (overlay mode, SPY Fluorescence Mode, and color segmented fluorescence (CSF) mode) offered by the SPY-Phi system.

If lymphatic leakage was detected at one of the described time points, ligature or clip closure of the leak was performed until no further lymphatic leakage could be demonstrated. Notably, no surgical drain was placed during the surgical procedure.

For screening of perirenal fluid, patients were screened by ultrasound examination during the three-weeks hospital stay on a weekly basis. After patient discharge, a weekly follow-up with routine ultrasound examination of the transplanted kidney was performed, which was accompanied by an MRI performed 6 months after kidney transplantation, focusing on the visualization of perirenal fluid.

## Results

In all 21 consecutive kidney transplantations, intraoperative fluorescence lymphography could be established. The best visualization of the perivascular lymphatic vessels was achieved using the SPY-fluorescence mode at all time points (Fig. 1). The best correlation to macroscopic anatomy could be achieved using the CSF mode, facilitating the localization of potential leakage.

**Fig. 3:**
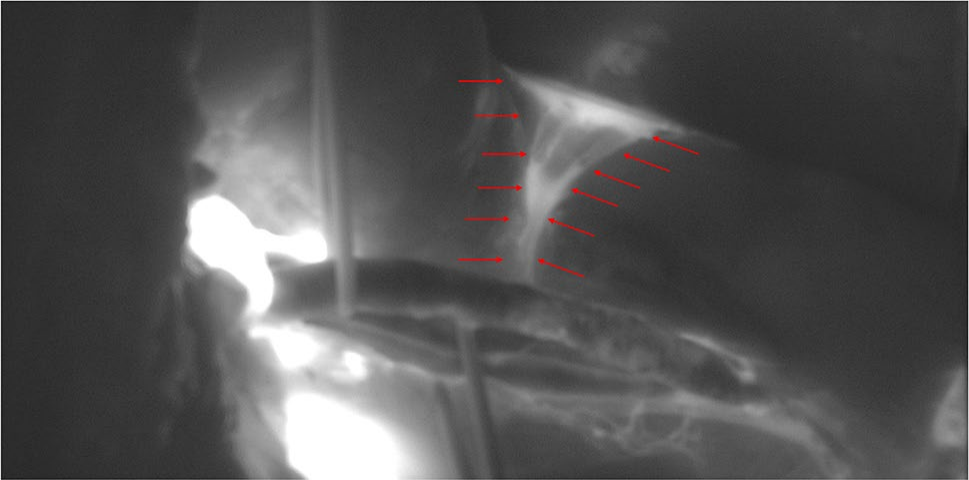
Intraoperative real time Lymphography showing lymphatic extravasation (red arrows) at t1

No side effects of the perivascular ICG injection were observed during the study period. Real-time lymphography revealed intact lymphatic vessels in 19 patients at all time points. Lymphatic leakage, in turn, was detected in two cases at t1 (Fig. 3). In these cases, lymphatic ligature and clip application were performed until no further leakage was observed on the real-time lymphography. In the subsequent measurements (t2 and t3) of these cases, no further lymphatic extravasation was observed.

During the hospital stay, weekly ultrasound examinations of the transplanted kidneys were performed. No fluid collection was documented in 19 cases. In 2 cases, postoperative hematoma surrounding the transplanted kidney was diagnosed. No further clinical action was needed in these cases, and further sonographic examinations showed that the hematoma had declined in size until full adsorption. After a follow-up period of 6 months, MRI of the transplanted kidney was performed. Six patients withdrew their declaration of consent regarding the final MRI examination and were alternatively examined by ultrasound. Thus, these examinations revealed no evidence of lymphocele formation in any of the 21 patients.

All 21 patients included in the study showed adequate function of the transplanted graft, with a mean serum creatinine level of 1,58 mg/dl at discharge. In two patients diagnosed with ADPKD, a simultaneous laparoscopic cystnephrectomy was performed before transplantation to create sufficient space for the transplanted organ. Seven kidney transplants showed multiple arteries (6, *n* = 2; 1, *n* = 3) that were inserted separately into the iliac artery. All patients were treated with triple immunosuppressive therapy comprising of a calcineurin inhibitor (CNI; tacrolimus), antiproliferative drugs (MMF), and corticosteroids. There was no delayed graft function documented in the study group, and no patient had a postoperative complication (Clavien-Dindo > 3a). In four cases, kidney transplant biopsy was performed because of deterioration of renal function during the hospital stay and revealed borderline suspicion of acute T-cell-mediated rejection. The patients were treated with steroid pulse therapy.

## Discussion

This prospective, controlled pilot FLIKT-study at hand demonstrated that intraoperative indocyanine green (ICG)-guided lymphography is a safe and feasible method for real-time visualization of lymphatic vessels during kidney transplantation, with great potential to reduce the risk of postoperative lymphocele formation. This technique was first described by Ietto et al. in 2016. In their case report, the authors demonstrated that ICG injection can be used to intraoperatively visualize lymphatic tissue during kidney transplantation [16]. Our current study corroborates and expands on these findings in case series of 21 living donation kidney transplantation recipients. In addition, Boni et al. recently described the feasibility of ICG-guided retroperitoneal lymph node dissection in urological oncology, demonstrating that fluorescence guidance enables clear visualization of retroperitoneal lymphatic structures and facilitates precise dissection [19]. Their work highlights the growing relevance of ICG fluorescence for intraoperative lymphatic mapping beyond transplantation and confirms the broader applicability of this technical principle to complex retroperitoneal surgery. In our study, ICG injection provided clear intraoperative imaging of lymphatic structures in all 21 cases, enabling preservation during iliac dissection. Importantly, lymphatic leakage could be identified and ligated effectively in two patients, thereby possibly preventing postoperative complications. Notably, no lymphoceles were observed during the six-month follow-up period, supporting the effectiveness of this technique.

Our findings are consistent with previous reports indicating that most lymphoceles originate from the unligated iliac lymphatics rather than from the transplanted graft itself [6, 20]. ICG fluorescence lymphography provides real-time imaging and adds an intraoperative safeguard that complements meticulous surgical techniques. This is particularly relevant, given that conventional approaches largely rely on visual assessment and experience, which may not reliably identify all lymphatic structures. The ability to detect and manage lymphatic leakage directly during surgery represents a paradigm shift toward prevention rather than treatment of lymphoceles.

The incidence of lymphoceles varies widely in the literature, ranging from 6 to 40%, depending on patient risk factors, immunosuppressive regimens, and surgical techniques [2, 3, 5]. In our cohort, the complete absence of lymphocele formation was notable and may reflect the added value of ICG lymphography.

A major strength of the study design was the systematic imaging approach at multiple defined intraoperative time points, which ensured reproducibility and enabled the detection of dynamic changes such as leakage after vascular dissection. Using a handheld near-infrared imaging system was practical and could be easily integrated into the surgical workflow with no adverse reactions observed. This highlights the technical feasibility and safety of this method in transplant settings.

However, some limitations of this study must be acknowledged. First, as safety and everyday feasibility were the primary study goals, the relatively small sample size, missing case controls, and single-center design of our pilot trial, in turn, limited the generalizability of these results. Second, the study only included living-donor kidney transplants, which are often associated with shorter operative times and potentially lower complication rates than deceased-donor transplantations. Third, six patients declined the final MRI follow-up, relying instead on ultrasound. Although ultrasound is sensitive, it may be less accurate than MRI in detecting small or asymptomatic lymphoceles. Although the six-month follow-up period is adequate to capture the majority of symptomatic lymphoceles (which peak around six weeks post-transplant), late occurrences may not be fully excluded.

Therefore, future studies should include larger multicenter cohorts, stratifying patients according to known lymphocele risk factors, such as immunosuppressive regimen, BMI, and surgical complexity. Randomized controlled trials comparing standard care with and without ICG lymphography could provide definitive evidence of its preventive benefit. Our pilot-FLIKT-Trial is an important step in paving the way for these complex multi-center trials and may help to guide their study design. In this context, cost-effectiveness analyses are required to determine whether the reduction in lymphocele-related interventions offsets the additional costs of fluorescence imaging systems.

In summary, this pilot study provides compelling evidence that intraoperative ICG-guided lymphography is a valuable addition to kidney transplantation surgery. By enabling real-time identification and preservation of lymphatic vessels, this technique has the potential to reduce postoperative morbidity, improve graft outcomes, and establish a new standard of care in transplant surgery.
